# Haemophilus influenzae Bacteremia and Pneumonia: A Case Report

**DOI:** 10.7759/cureus.49395

**Published:** 2023-11-25

**Authors:** Abraham Tanousian, Muhammad R Bajwa, Nina Aghakhani

**Affiliations:** 1 Internal Medicine, California Health Sciences University College of Osteopathic Medicine, Clovis, USA; 2 Internal Medicine, Kaiser Permanente Fresno Medical Center, Fresno, USA

**Keywords:** covid-19, pneumonia, bacteremia, invasive bacterial disease, haemophilus influenzae

## Abstract

This study reports a case of respiratory failure and pneumonia attributed to infection from a confirmed case of *Haemophilus influenzae* in a patient with past medical history of interstitial lung disease following a COVID-19 infection. An 88-year-old man with significant past medical history of interstitial lung disease (ILD) and self-catheterization due to benign prostatic hyperplasia (BPH) presented to the ED with shortness of breath and cough. Examination revealed reduced respiratory effort and scattered rhonchi throughout the lung fields. Urine cultures were positive for extended spectrum beta-lactamase (ESBL) *Escherichia coli*. In addition, blood cultures and chest X-ray findings confirmed a case of *H. influenzae* bacteremia and pneumonia. The following case highlights the unusual finding of invasive *H. influenzae* disease and corresponds with the data provided by the Active Bacterial Core surveillance supported by the Centers for Disease Control and Prevention (CDC).

## Introduction

Infection with *Haemophilus influenzae* is a fairly rare phenomenon in the current clinical landscape [1]. Historically, *H. influenzae* infections were predominantly seen in the pediatric population, with the most pathogenic strain being serotype b (Hib). By the late 1980s, the advent of the first *H. influenzae* vaccines licensed for use in infants aged greater than two months drastically reduced the incidence rates of invasive *H. influenzae* infections [1]. Although serotype b is widely recognized as the most virulent serotype, it is important to note that other serotypes and non-typeable *H. influenzae* have also been linked to invasive diseases. These invasive conditions include meningitis, epiglottitis, septic arthritis, cellulitis, pericarditis, endocarditis, and osteomyelitis [2]. Consequently, the prompt detection and management of *H. influenzae* plays a vital role in preventing substantial morbidity and mortality, but it remains low on most practicing clinicians' list for respiratory failure due to an infectious process. 

Clinical presentations for respiratory failure due to pneumonia are highly variable depending largely on the type of strain, bacterial load, patient age, and comorbidities present during admission [2,3]. Common clinical findings include but are not limited to fever, shortness of breath, cough with and without sputum production, respiratory distress, and mental status changes [3,4]. Physical exam supports these findings with the presence of decreased breath sounds, crackles on auscultation, dullness on percussion, tactile fremitus, and decreased oxygen saturation on pulse oximetry [4]. The typical workup for respiratory failure and sepsis secondary to pneumonia starts with a complete blood count with differential alongside anteroposterior and lateral chest radiographs. If clinical suspicion is high for septicemia or lung abscess, CT of the chest with blood cultures are ordered to view the degree of infection. In the case of *H. influenzae*, real-time PCR typing may be obtained; however, in most clinical instances, this step is omitted due to similar treatment modalities being utilized [4].

## Case presentation

We present the case of an 88-year-old male with past medical history of hypertension (HTN), obstructive sleep apnea (OSA) on continuous positive airway pressure (CPAP), history of interstitial lung disease (ILD) secondary to COVID-19 infection, and benign prostatic hyperplasia (BPH) with self-catheterization presenting to the ED with chief complaints of dry cough and shortness of breath lasting two weeks. Onset of symptoms followed exposure to his acutely ill grandchildren. The patient stated that he is typically able to ambulate around his home with a cane, but has since been bed-bound. The patient reported symptoms having begun to worsen 24 hours prior to ED admission. In an attempt to alleviate worsening symptoms, he (re)started home oxygen therapy up to 4-5 L of O_2_ per minute via nasal cannula with no success. Most recent at-home SpO_2_ reading was 85%, prompting the patient's daughter to seek emergency care. He denied associated symptoms, including chest pain, hemoptysis, diaphoresis, edema, nausea, vomiting, diarrhea, dysuria, and hematuria. He also denied recent contact with chemicals, irritants, or allergens.

**Figure 1:**
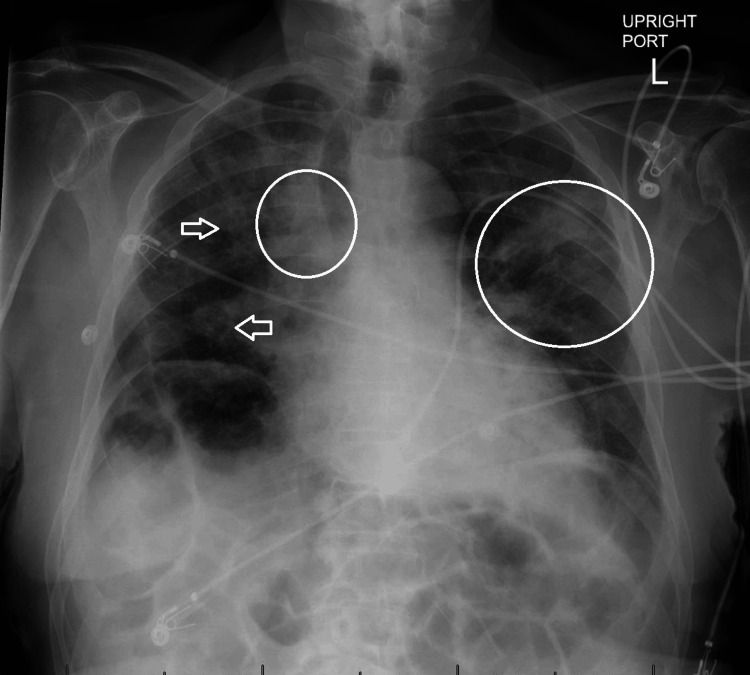
Anteroposterior chest radiograph identifying the location of consolidations (circles) and bronchial wall thickening (arrowheads).

The patient presented afebrile, normotensive, hypoxic with an oxygen saturation of 89 on admission, mild tachycardia with a heart rate peaking at 111, and a respiratory rate that generally fluctuated in the low 20s. Upon general inspection, the patient appeared ill, but he was alert and oriented to self, time, place, and purpose. The head, eyes, ears, nose, and throat (HEENT) exam noted intact extraocular eye movements with anicteric sclera, moist mucous membranes, and absence of cervical/supraclavicular lymphadenopathy. Regular rate and rhythm of S1/S2 with no murmurs, rubs, or gallops upon cardiac auscultation were noted. Scattered rhonchi auscultated throughout bilateral upper lung lobes, with right-sided prominence and no wheezes or rales bilaterally. No peripheral edema of bilateral lower extremities and no cyanosis or clubbing of digits were observed. Radial and carotid pulses were +2 bilaterally. ECG revealed the presence of a previously noted right bundle branch block (RBBB). Chest radiograph was alarming for bilateral areas of consolidation in both upper lobes with bronchial wall thickening more prominent on the right (Figure 1). It was difficult to discern whether these findings were related to CHF or pneumonia, but the asymmetric nature of the infiltrates are pathognomonic of an infectious process. Labs showed leukocytosis of 29.8 x 10^9^ cells/L (reference range = 4.5 to 11 x 10^9^ cells/L) with 90% neutrophils. The patient was empirically started on intravenous (IV) Zosyn and was admitted to inpatient services for the treatment of respiratory failure secondary to sepsis. 

During his first admission day, the patient developed atrial fibrillation with rapid ventricular response, at which point he was upgraded to a telemetry unit. He was treated with both oral and IV metoprolol. Urine culture was positive for >100,000 colonies/mL of *Escherichia coli* with a multitude of resistances, at which point the prophylactic Zosyn was switched to ertapenem by approval of the infectious disease consult. He was also on a breathing treatment, which included bronchodilators and supplemental oxygen for the symptomatic relief of shortness of breath. The first set of blood cultures were positive; as a result, a second set was ordered to rule out contamination. Day two saw the placement of a midline by the RN due to anticipation of two weeks of IV antibiotic regimen. The patient’s shortness of breath was subjectively better and he was clinically improving. On day three, the pending blood cultures for Gram stain and Kirby-Bauer testing were positive for *H. influenzae* with widespread susceptibility; no changes were made to the antibiotic regimen as ertapenem provided coverage for both sources of infection. Day four saw no changes in the patient’s medical plan, and he continued to be on 2 L of oxygen by nasal cannula. After continued clinical improvement with inpatient treatment, he was discharged home on day five in a stable condition with a two-week regimen of IV ertapenem and at-home oxygen for chronic respiratory failure. A two-week post-discharge telephone appointment with a primary care physician revealed a favorable outcome. The patient was feeling well and had begun transition to oral antibiotics. Further telephone advice visits with his primary care physician noted to have continued clinical improvement.

## Discussion

The following case demonstrated a rare presentation of confirmed *H. influenzae* bacteremia and pneumonia in an elderly patient with significant past medical history of interstitial lung disease following a COVID-19 infection. Of note, the laboratory that confirmed the case of *H. influenzae* did not identify the serotype/species. Taking into account the data provided by the Active Bacterial Core surveillance group, which is supported by the Centers for Disease Control and Prevention (CDC), there can be an argument made for a non-typeable strain infection. Data collected from both 1999-2008 [5] and 2009-2015 [6] illustrate that the vast majority of invasive *H. influenzae* infections are due to non-typeable strains. From 2009 to 2015, the incidence rate of non-typeable *H. influenzae* invasive disease, regardless of age, was 1.22 cases per 100,000 cases per year, which was significantly more than non-B serotypes (0.45) and serotype B (0.03) [6]. In addition, in regard to the patient in our case, the incidence rate of non-typeable invasive disease, in patients greater than age 65, was 4.99 cases per 100,000 cases per year (non-B serotypes 1.27, serotype B 0.04). With a case fatality rate of 21.3% for non-typeable strains and 19.9% for all strains, patients above the age of 65 are at an increased risk, so careful clinical assessment and testing is paramount in preventing adverse outcomes [6].

Interestingly, there is a shift in the incidence rates when taking into account the COVID-19 pandemic. The same group that compiled the data above tracked the incidence rates of invasive bacterial diseases (IBDs) from March 1 to December 30 2020. Compared with expected incidents, the incidence of invasive *H. influenzae* disease was 60% lower [7]. Researchers believed that this trend was explained due to a combination of improved personal protective equipment (PPE) use in hospital settings on top of the increased use of non-pharmaceutical interventions (NPIs). The use of NPIs was quantified using the Oxford COVID-19 Government Response Tracker (OxCGRT) stringency index, which quantified how strict government regulations were in regard to public gathering, travel, and school/workplace changes [7]. May 11, 2023 marked the end of the Public Health Emergency issued by the federal government. As such, it is reasonable to suspect that the changes in epidemiology will also wane and most likely revert to the expected incidence rates outlined prior to the pandemic. This reinforces the importance of continued public health practices to educate elderly patients with comorbidities on the dangers of bacterial and viral exposure to reduce the risk of life-threatening infections.

## Conclusions

*H. influenzae *is often overlooked in elderly patients presenting with acute respiratory failure due to pneumonia. Patients with a significant history of lung pathology and continued self-catheterization are at higher risk of developing invasive bacterial disease. We hypothesize that the patient’s direct contact with his grandchildren showing signs and symptoms of upper respiratory tract infection played a key role in the disease etiology. As in this case, past medical and social history hold key clues to guide physician diagnosis.
